# Incidental Discovery of a Urethral Leiomyoma: A Rare Case of an Asymptomatic Benign Tumor With Successful Surgical Management

**DOI:** 10.7759/cureus.84662

**Published:** 2025-05-23

**Authors:** Tariq Abdul Hamid, Thureya Binashour, Mohamad Motaz Al Masri, Kais Kotiesh, Fariborz Bagheri

**Affiliations:** 1 Surgery, Dubai Hospital, Dubai, ARE; 2 Urology, Dubai Health, Dubai, ARE; 3 Urology, Dubai Hospital, Dubai, ARE

**Keywords:** benign mesenchymal tumor, benign urethral mass, case report, female urethral tumor, histopathology (hp), magnetic resonance imaging (mri), open surgical excision, smooth muscle neoplasm, urethral leiomyoma, urethral mass

## Abstract

Urethral leiomyomas are uncommon benign tumors arising from urethral smooth muscle. These tumors predominantly occur in women of reproductive age and are frequently asymptomatic or incidentally detected. We present the case of a 33-year-old woman with a small, symptomless mass near the urethral meatus. MRI identified a 1.7 × 1.4 cm lesion in the distal urethra, and subsequent cystoscopy-guided surgical excision confirmed the diagnosis of urethral leiomyoma. The patient recovered well postoperatively, and histopathological examination verified complete tumor removal. Given the low recurrence risk after excision, surgical resection remains the treatment of choice. This case emphasizes the importance of including urethral leiomyoma in the differential diagnosis of urethral masses and demonstrates the utility of MRI and surgical intervention in effective management.

## Introduction

Primary urethral leiomyoma is a rare benign entity with only about 100 cases reported in the literature [1]. It is a benign mesenchymal tumor originating from the smooth muscle of the urethra [2]. It develops mainly from the circular fibers of the smooth muscle, with the kidney capsule being the most common location in the genitourinary system [3]. Urethral leiomyoma often occurs in female patients aged 20 to 50 years, while it is exceptionally rare in males. The most common type of leiomyoma in the human body is uterine leiomyoma, but it can also occur in uncommon sites such as the external genitalia, ovaries, urethra, or bladder [4]. Often diagnosed on clinical examination or as an incidental finding, MRI proves to be a helpful tool in localizing urethral leiomyoma [3]. Surgical excision is the preferred treatment, with complete excision of the lesion resulting in few reports of recurrence or metastasis. This case report describes a 33-year-old female with an incidental asymptomatic urethral leiomyoma managed surgically with a good postoperative course.

## Case presentation

A 33-year-old nulliparous woman presented to the urology outpatient clinic after she incidentally discovered a lump near her urethral opening while wiping her external genitalia. She denied any associated urinary symptoms, including dysuria, frequency, urgency, incontinence, or hematuria. There was no history of trauma, urinary tract infections, bleeding per urethra, or prior instrumentation. Her medical, surgical, and gynecological histories were unremarkable, and she was not on any regular medications. She reported regular menstrual cycles and had no known hormonal imbalances.

On physical examination, a well-defined, reddish, oval-shaped mass was noted protruding approximately 0.5-1 cm from the posterior urethral meatus and extending proximally by a similar dimension. The lesion appeared firm yet mobile on gentle palpation, with mild tenderness and no active bleeding or ulceration. The external urethral meatus was clearly identifiable anterior to the mass but was noted to be moderately compressed by the lesion. No signs of infection, such as erythema or discharge, were observed. A digital vaginal examination revealed no deep extension into the anterior vaginal wall or palpable masses elsewhere.

**Figure 1:**
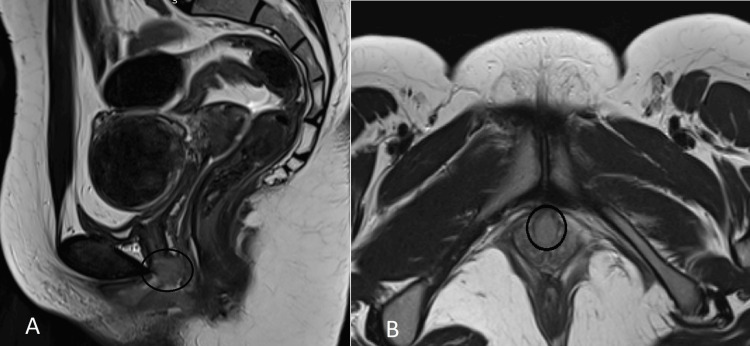
(A) Sagittal T2-weighted image demonstrating a well-defined, enhancing lesion (circled) located in the distal urethra, extending posteriorly to abut the anterior vaginal wall. (B) Axial T2-weighted image showing the same lesion (circled), centered in the distal urethra and predominantly intraluminal, without evidence of deep pelvic extension or invasion into adjacent structures.

A pelvic MRI scan was performed for further characterization of the mass. Imaging revealed a well-circumscribed, irregularly marginated, enhancing lesion measuring approximately 1.7 × 1.4 cm located in the distal urethra. The lesion was predominantly intraluminal, with posterior extension abutting the anterior vaginal wall, but without evidence of deep tissue invasion or extension into adjacent pelvic organs. The proximal two-thirds of the urethra appeared normal, and there were no features suggestive of malignancy, such as infiltration, necrosis, or lymphadenopathy (Figure 1).

**Figure 2:**
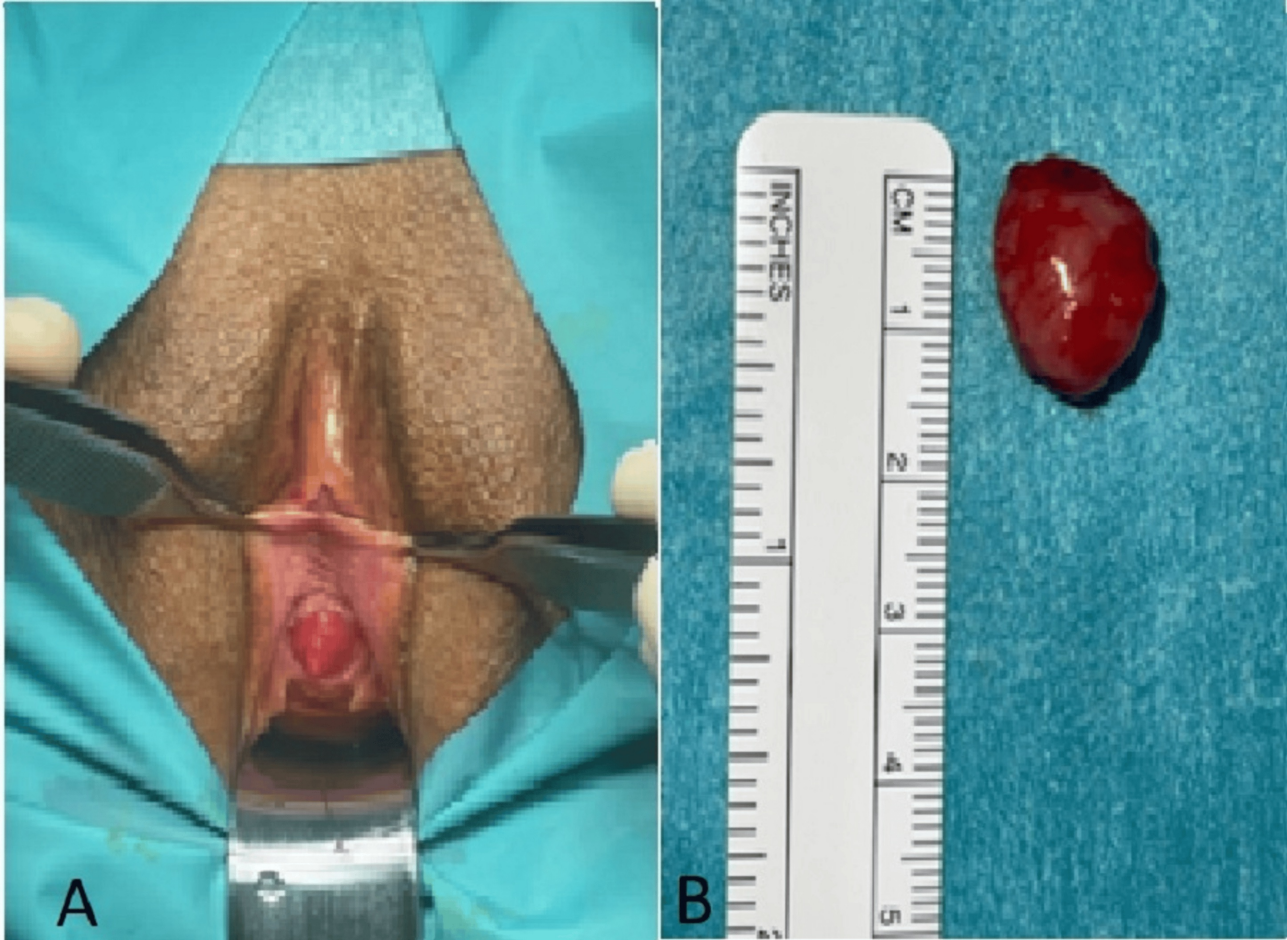
(A) Intraoperative view showing a well-circumscribed, reddish, oval-shaped mass protruding from the posterior aspect of the distal urethral meatus, with visible urethral orifice anterior to the lesion. (B) Gross specimen of the excised urethral mass measuring approximately 1.5 × 1.5 cm, consistent with a urethral leiomyoma, shown alongside a ruler for size reference.

Cystourethroscopy under anesthesia was performed, which confirmed the presence of a solitary, smooth, mucosa-covered mass arising from the anterior urethral wall. The mass did not obstruct the lumen completely, and the proximal urethra and bladder appeared normal. Given the location and size, open surgical excision was undertaken. The mass, measuring approximately 1.5 × 1.5 cm intraoperatively, was carefully mobilized using traction sutures and excised in its entirety with a margin of surrounding tissue to ensure complete removal (Figure 2). Hemostasis was achieved, and the urethral mucosa was reconstructed. A Foley catheter was inserted at the end of the procedure to allow for healing and was left in place for three weeks.

The patient’s postoperative recovery was uneventful. She was discharged on the first postoperative day with appropriate analgesia and catheter care instructions. At the three-week follow-up visit, the catheter was removed, and her voiding pattern was assessed. Post-void residual volume was minimal (30 mL), and she reported no urinary complaints or discomfort. The surgical site had healed completely, and there were no signs of recurrence or complications.

**Figure 3:**
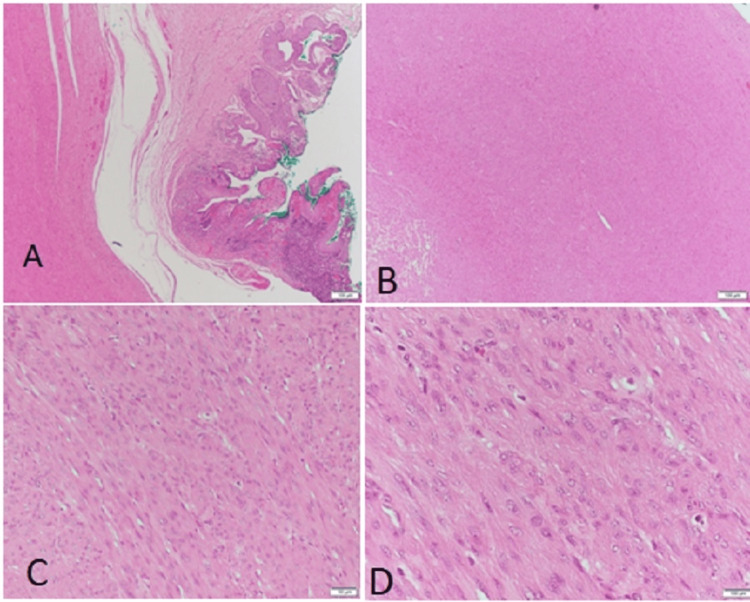
(A) Low-power view (4×) showing an ulcerated urothelium overlying a subepithelial spindle cell lesion with moderate chronic inflammatory infiltrate. (B) Low-power view (10×) highlighting a well-circumscribed spindle cell neoplasm. (C) Medium-power view (20×) demonstrating interlacing bundles of spindle cells with uniform architecture. (D) High-power view (40×) showing intersecting fascicles of monotonous spindle cells with indistinct cytoplasmic borders, eosinophilic cytoplasm, and elongated, cigar-shaped nuclei with tapered ends and inconspicuous nucleoli. No evidence of atypia or mitotic activity is seen.

Histopathological examination of the excised specimen revealed interlacing bundles of benign smooth muscle cells without atypia or mitotic activity, consistent with a diagnosis of urethral leiomyoma. No evidence of malignancy or necrosis was noted (Figure 3). The diagnosis was confirmed as a completely excised urethral leiomyoma. The patient was counseled on the benign nature of the lesion and the low likelihood of recurrence, but was advised to follow up in six months with imaging surveillance at intervals.

## Discussion

Urethral leiomyoma represents a rare benign mesenchymal tumor originating from urethral smooth muscle fibers, with fewer than 100 cases documented in the literature [1,2]. While historically considered exceptionally uncommon, recent institutional reviews suggest under-diagnosis due to frequent asymptomatic presentations, identifying seven additional cases in a single-center study [5]. These tumors predominantly affect reproductive-aged women (mean age: 41 years [1,6]), with strong immunohistochemical evidence of hormonal dependence, i.e., estrogen/progesterone receptor positivity in 78% of cases, correlating with clinical observations of pregnancy-associated growth and postmenopausal regression [6,7]. Anatomically, they arise most frequently from the posterior proximal urethra [3] and are classified as deep soft tissue leiomyomas, distinct from cutaneous or vascular subtypes [4,8].

Clinically, urethral leiomyomas exhibit position-dependent manifestations: lesions at 12 or 6 o’clock positions typically cause obstructive symptoms (e.g., dysuria, retention) [1], while lateral tumors provoke irritative voiding complaints [1,9]. Approximately 25% remain asymptomatic and are detected incidentally [4], as exemplified by our patient’s self-palpated mass. The diagnostic workup hinges on multimodal assessment: pelvic MRI optimally defines tumor dimensions, local invasion (e.g., anterior vaginal wall abutment [3]), and excludes malignant features (e.g., necrosis) [7]. Cystoscopy confirms intraluminal involvement, while histopathology (spindle cells with eosinophilic cytoplasm, absent atypia [7]) and immunohistochemistry (desmin+, actin+) provide definitive diagnosis [7,10]. Key differentials span benign entities (urethral diverticulum, paraurethral cyst) to malignancies (leiomyosarcoma, squamous cell carcinoma) [4,9].

Surgical excision remains the gold standard, with the approach tailored to the tumor location and size. Transurethral resection is advocated for distal lesions <2 cm (100% success rate in reported series [11]), whereas proximal or large (>3 cm) masses often require open excision with urethral reconstruction to preserve continence [4,5]. Our patient’s distal 1.7 cm lesion facilitated complete surgical resection, aligning with reported outcomes of zero recurrences at five-year follow-up when complete base excision is achieved [5]. Although postoperative complications (e.g., urethrovaginal fistula, stress incontinence) are rare (<5% [1]), meticulous surgical technique is paramount. Adjuvant hormonal therapy (gonadotropin-releasing hormone agonists) may reduce tumor volume preoperatively in select cases [3,10], while annual MRI surveillance for three years post-resection is recommended, given delayed recurrences in isolated reports [5]. Notably, no malignant transformations have been documented [5,7], underscoring the benign nature of this entity.

## Conclusions

This case contributes to the limited body of literature on urethral leiomyomas, emphasizing the importance of considering this rare entity in the differential diagnosis of urethral masses. As evidenced by the literature, the true incidence may be higher than previously reported due to asymptomatic presentations. Comprehensive clinical and radiological evaluations, coupled with surgical intervention, remain the cornerstone of effective management. Immunohistochemical analysis for hormone receptors should be considered to guide long-term surveillance, particularly in reproductive-aged women. Although there have been very few cases of reported recurrences in the literature after excision, it still underlines the significance of surveillance to ensure long-term efficacy.
